# Brains of Native and Alien Mesocarnivores in Biomonitoring of Toxic Metals in Europe

**DOI:** 10.1371/journal.pone.0159935

**Published:** 2016-08-11

**Authors:** Elzbieta Kalisinska, Natalia Lanocha-Arendarczyk, Danuta Kosik-Bogacka, Halina Budis, Joanna Podlasinska, Marcin Popiolek, Agnieszka Pirog, Ewa Jedrzejewska

**Affiliations:** 1 Department of Biology and Medical Parasitology, Pomeranian Medical University, Szczecin, Poland; 2 Department of Health Education, University of Szczecin, Szczecin, Poland; 3 Department of Environmental Management and Protection, Western Pomeranian University of Technology, Szczecin, Poland; 4 Department of Parasitology, Institute of Genetics and Microbiology, University of Wroclaw, Wroclaw, Poland; 5 Department of Invertebrate Systematics and Ecology, Institute of Biology, Wroclaw University of Environmental and Life Sciences, Wroclaw, Poland; 6 Warta Mouth National Park, Chyrzyno, 1, Poland; University of Louisville School of Medicine, UNITED STATES

## Abstract

Mercury (Hg), lead (Pb) and cadmium (Cd) are involved in mammalian brain damage. However, little is known about Pb and Cd brain levels in wildlife that reflect the geochemical background. The aims of the study include the estimation of Hg, Pb and Cd concentrations, and the determination of relationships between these elements in the brains of 94 mesocarnivores. Road-killed or hunted animals were obtained from north-western Poland near the Polish-German border. The investigation covered the native Eurasian otter *Lutra lutra*, badger *Meles meles*, pine marten *Martes martes*, beech marten *M*. *foina*, European polecat *Mustela putorius*, red fox *Vulpes vulpes*, and alien species: feral and ranch American mink *Neovison vison*, raccoon *Procyon lotor* and raccoon dog *Nyctereutes procyonoides*. Depending on the diet and environmental pollution, the carnivore brains accumulated toxic metals in varying amounts. The highest median Hg levels (in mg/kg dry weight, dw) were found in the piscivorous Eurasian otter and feral mink (2.44 and 3.96), Pb in the omnivorous raccoon (0.47), while Cd in minks (~0.06). We indicated that Pb-based ammunition is a significant source of the element in scavengers from hunting area, and we also found a significant correlation between Pb and Cd levels in the fox brain. Finally, this study is the first to suggest background levels for brain Pb and Cd in mesocarnivores (<0.50 and <0.04 mg/kg dw, respectively).

## Introduction

Heavy metals present in the environment are released from natural (volcanic activity, erosion of ore-bearing rocks) and anthropogenic sources (burning fossil fuels, mining and processing of metal ores, mechanical, chemical, automotive industries, transport and agriculture). An increasing amount of heavy metals began to be introduced into the natural cycle as a result of industrial development, beginning in Europe with the Industrial Revolution (17^th^/18^th^ centuries) and strongly intensifying mainly in the northern hemisphere after World War II [1,2].

Heavy metals, i.e. those with density exceeding 4–5 g/cm^3^ [3,4], are divided into two groups–essential (components of structural proteins, enzymes, hormones) and non-essential (xenobiotics), which do not have any biological function [5,6]. After reaching certain levels in the body, they cause disturbances at molecular, cellular, tissue and organ levels, and sometimes lead to serious illnesses and even to death. Three non-essential metals, mercury (Hg), lead (Pb) and cadmium (Cd), are primary toxic heavy metals under frequent study, known to affect mammals in different ways [7–10]. Usually, these metals reach warm-blooded vertebrates via food, with inhalation also playing a significant role in areas with high air pollution [11,12].

In mammals, gastrointestinal absorption of Hg, Pb and Cd is influenced by the physiological state of the exposed animal (including age, fasting status and content of nutritionally essential elements) as well as the physico-chemical properties of the toxic metals and their bioavailability. Generally, inorganic forms of Hg, Pb and Cd are poorly absorbed in the mammalian alimentary tract (from <3% to 15–20%) but more than 90% of methylmercury (MeHg) is assimilated [7,13,14]. Methylmercury is produced by bacteria present in aquatic sediments (and to a lesser degree in wetland soils) in biomethylation processes. It is then able to enter the aquatic food chain where it undergoes significant biomagnification, with long-lived predatory fish showing the highest concentrations. Nearly all mercury in fish is MeHg, the main source of exposure of piscivorous mammals [15,16].

Although it appears that Pb and Cd are not biomagnified, all three of the studied metals are bioaccumulated in the tissues of warm-blooded vertebrates [11,12,15,17,18]. The brain is most vulnerable to the effects of Hg and Pb, which is well documented in humans and numerous pre- and postnatal animal experiments. Both these neurotoxins pass through the brain-blood and placental barriers, and during pregnancy can act as teratogens [7,19,20].

Characteristic Hg-related changes in the brain involve structural degeneration (especially in the occipital cortex and cerebellum) to cause visual, cognitive and neurobehavioral deficits. The central and peripheral nervous systems of wild mammals are the primary targets for MeHg toxicity as this form of Hg is readily absorbed from the diet and can easily pass the blood–brain barrier [19,21]. When it comes to wild terrestrial mammals, data on the tissue concentrations of Hg and MeHg toxicity are more plentiful for semiaquatic piscivorous carnivores (such as the North American river otter *Lontra canadensis*, Eurasian otter *Luta lutra*, and American mink *Neovison vision*) than for other species, and usually concern the liver and kidneys [22–25].

The toxic effects of Pb in the brain include reduced weight of the organ, increased cerebral pathology, lack of coordination, impaired motor skills, convulsions, impaired visual discrimination and learning, abnormal social behavior, increase in aggression. Lead poisoning results in reproductive impairment, increased fetal deaths and abortions, reduction of survival and longevity [12,14].

Cadmium may induce tumors in animals and humans, particularly in the kidney [8]. However, mammals exposed to Cd also reveal disturbances in the function of the nervous system, with symptoms including olfactory dysfunction, slowing of vasomotor functioning, learning disabilities, and behavioral disturbances [26–28].

Impaired vision, olfactory disturbances and learning ability connected with influence of Hg, Pb and Cd could be life-threatening to wild animals given that such dysfunctions could significantly impair their ability to catch prey, resulting in malnutrition, increased susceptibility to diseases, or reduced reproductive success. Despite the proven negative effects of Hg, Pb and Cd on the brains of mammals, there is a striking scarcity of papers on the concurrence and levels of these metals in wild mammals, including carnivores. We have found only one such paper, on the Javan mongoose *Herpestes javanicus* from Japan [29]. The greatest number of papers on the brains of wild carnivores concern just Hg contamination, and the most frequently studied animals were the (almost exclusively North American) river otter and American mink, and sporadically the raccoon *Procyon lotor*, Egyptian mongoose *Herpestes ichneumon*, red fox *Vulpes vulpes*, and Polar bear *Ursus maritimus* [22,30–37]. Concentrations of Pb and Cd in the brains of urban dogs and cats from Poland were reported by Michalska et al. [38] and Soltysiak et al. [39].

In ecotoxicological studies on mammalian soft tissues, the liver and kidneys are most commonly selected for analysis, as this is where Hg, Pb and Cd reach the greatest concentrations. In addition, those organs play an important role in detoxification. In comparison, the levels of toxic metals in the brain are much lower, yet they cause more serious consequences in the functioning of the central nervous system, including behavioral changes in individuals and whole communities. With regard to humans, it is important to control the concentration of toxic metals in the edible content of domestic and hunted animals, especially in meat. In this regard, researchers have focused primarily on large hoofed herbivorous mammals [40,41]. However, considering the health status of the whole terrestrial ecosystem, omnivorous and carnivorous mammals should also be taken into account. Top predators and scavengers, such as mid-sized and large carnivores, play important roles and are essential for the functioning of ecosystems [42,43]. Because of their position in the trophic web, these mammals are vulnerable to the bioaccumulation of pollutants, including toxic heavy metals [15,23,44,45].

Among other things, carnivores differ in body weight, metabolism rate, habitat, home range and diet. Among the terrestrial animals, the most abundant are small and midsized species (<15 kg), often collectively termed ‘mesocarnivores’. These species far outnumber the larger carnivores in species richness and are much more diverse in their behavior and ecology [43]. Some mesocarnivores are piscivores (such as otters and the American mink), while in the canid subgroup (e.g. foxes–genera *Vulpes* and *Urocyon*, raccoon dog *Nyctereuts procyonoides*) small mammals, birds and carrion form an important part of their diet. Badgers prefer earthworms, and typical terrestrial mustelids (genera *Mustela* and *Martes*) feed on plant and animal material [46].

Therefore, it can be expected that the animals coming from the same area will accumulate toxic metals in differing amounts, depending–among other things–on the diet and degree of contamination of the environment in which they live. Many species of mesocarnivores are game fur animals, some of them live near human habitations and have the status of synanthropic animals (including martens, foxes, raccoon *Procyon lotor*), some species have been domesticated (cat, dog, fox), while others are kept at fur ranches: red fox, American mink and raccoon dog, which sometimes run away and then live and reproduce in the wild. Some carnivores (intentionally or unintentionally) have been introduced into Europe and at present are common elements of the continental fauna, posing serious problems for protected areas [47]. A number of native and alien mesocarnivore species are game animals and often victims of road accidents. Thanks to this, their carcasses may be easily accessed for ecotoxicological studies [23,48,49].

The aim of this study then was to evaluate and compare the concentrations of Hg, Pb and Cd in the brains of nine carnivorous mammals from north-western Poland and to examine the relationships between the levels of these toxic metals. This determination of the comparative levels of the three toxic metals in the brains of a large group of mesocarnivores, diverse in ecology, and derived from a relatively a small area, is the first analysis of this kind not only in Europe but in the world.

## Materials and Methods

### Study area

**Fig 1:**
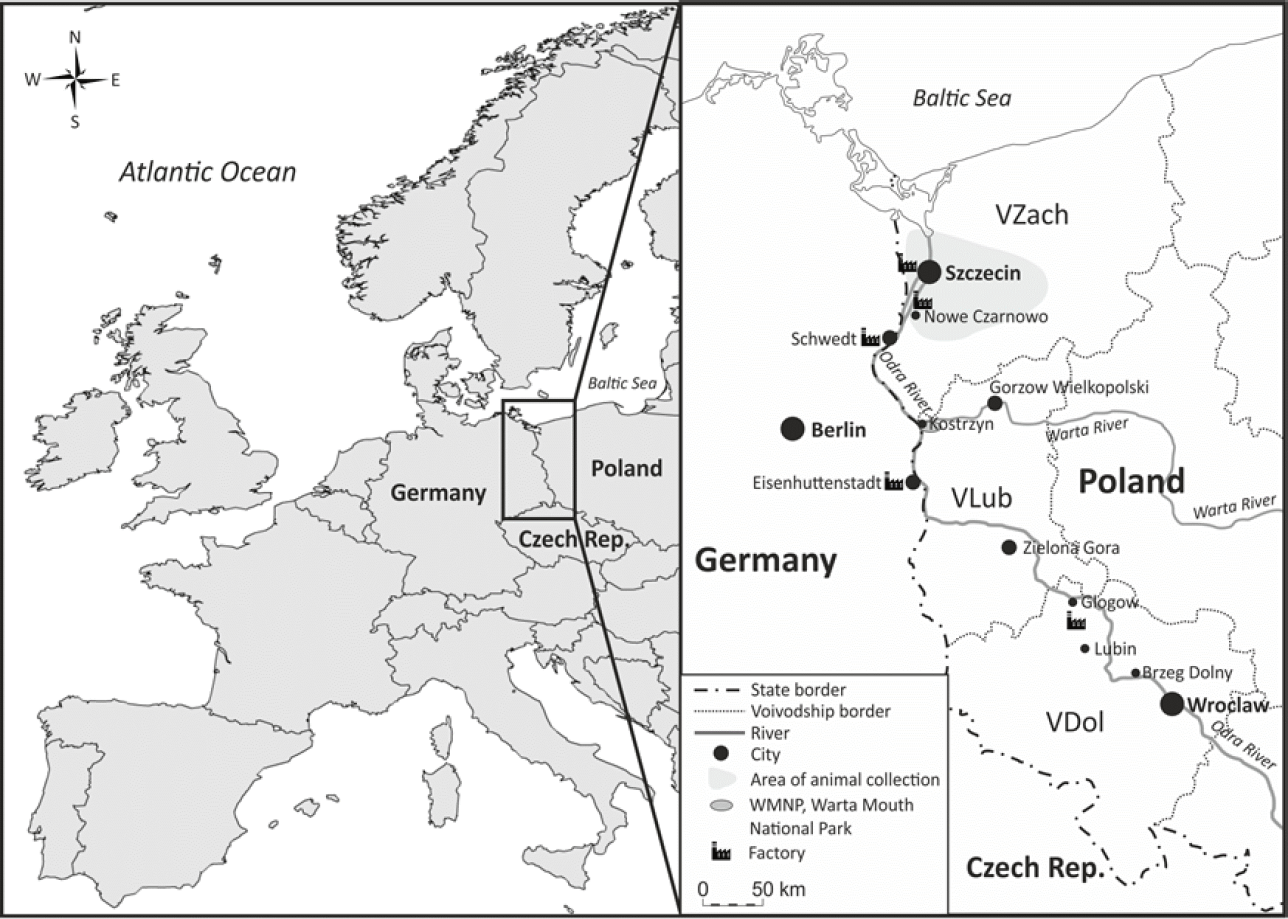
The area where the animals were collected is located at the Polish-German border. Winds in this part of Europe are mostly from south-west and organisms living in the area are exposed to emissions from both local and distant sources.

Material for the study came from two areas of north-western Poland: the western part of the Zachodniopomorskie Voivodship, VZach (1.72 million inhabitants, area of 22 892 km^2^, including forest 35%, arable land 49%, surface water 5.7%; capital—Szczecin), and the Lubuskie Voivodship, VLub (1.02 million inhabitants, area of 13 989 km^2^, including forest 49%, arable land 40%, surface water 1.7%; capital—Zielona Gora). The study areas are located in the basin of the Odra River, the second largest in the country (Fig 1).

The collection sites in VZach were scattered (Szczecin and nearby areas), and in VLub were limited to the area of the Warta Mouth National Park, WMNP. The WMNP covers ~80 km^2^ of the Warta River valley (the Warta is the largest tributary into the Odra River, the outlet located near the town of Kostrzyn, 617 river km, situated on the Polish-German border). Floodplains comprise about half of the park area and consist of mosaic ecosystems, very important for waterfowl [50].

In the north-western provinces of Poland, air emissions of Hg, Pb and Cd are low compared to the highly industrialized south of Poland. In the 2000s, emissions of Hg, Pb and Cd in VZach were 0.51, 13.67, 1.67 tonnes, respectively, and in VLub 0.12, 5.93 and 0.86 tonnes, i.e. from 0.8% to 4.2% of the national emissions of those metals [51]. In VZach, significant sources of air pollution are two coal power plants: "Pomorzany" in Szczecin and "Dolna Odra" in Nowe Czarnowo (Fig 1). In addition, the area of north-western Poland, with predominant SW winds, is exposed to air pollution from Germany, including an industrial center in Schwedt by the Odra river, with one of Germany's largest oil refineries.

The concentrations of heavy metals in agricultural soils and forests of north-western Poland normally correspond to the values observed in natural soils. In Poland, it is assumed that the natural content of Hg, Pb and Cd in uncontaminated soil amounts to 0.20, 18 and 0.22 mg/kg dw [52]. However, in some places, in the areas of anthropogenically polluted flood waters of the Odra and Warta rivers, and in air coming from industrialized areas, the levels of Hg, Pb and Cd in soils are significantly elevated, especially in topsoil of urban areas and grazing lands [53]. In Poland the main anthropogenic sources of heavy metals in the middle Odra River is Dolnoslaskie Voivodship with its nonferrous metallurgy (especially copper, between Lubin and Glogow) and German Eisenhuttenstadt, where major industrial enterprises are located, e.g., the steelworks of the Eisenhuttenstadt-Ost [54].

Pollution of the lower section of the Warta is mainly due to emissions from the cities of Gorzow Wielkopolski and Kostrzyn. Research shows that the modern sediments of the Odra and Warta have significantly elevated levels of heavy metals, including Hg, Pb and Cd, compared to the geochemical background (GB geochemical background) adopted in Poland and widely accepted probable effect levels (PEL), i.e. concentrations above which adverse effects are expected to occur frequently [54,55,56]. In Kostrzyn the concentrations of toxic metals in the sediments of the Odra investigated by Boszke et al. [56] were: Hg 0.25–1.49, Pb 21.2–42.8, Cd 3.73–4.62 (mg/kg dw). For comparison, the levels of GB and PEL were (mg/kg dw): <0.05 and 0.486 for Hg; 10 and 91.3 for Pb; <0.5 and 3.53 for Cd, respectively [57,58]. These toxic metals present in the aquatic environment of the Odra and Warta affect fish and other organisms living there. In individuals specimens of predatory fish caught within WMNP (2009), such as pike (*Esox lucius*) and asp (*Leuciscus aspius*), we found a Hg concentration 0.99 and 1.4 mg/kg dw, respectively (unpublished data).

### Material

This study involved 9 carnivore species, enlisted and characterized in Table 1. Eight of them can be found in the Polish list of game animals, while the European otter is partially protected (Journal of Law 2005, No 45, Item 433; Journal of Law 2014, Item 1438). All specimens collected for analysis were collected in accordance with applicable Poland law.

**Table 1:** General information on the studied mesocarnivores (VLub, Lubuskie Voivodship; VZach, Voivodship Zachodniopomorskie; n, number; PL, Poland: status and trend population; EU, European Union: status according to Habitats Directive 92/43/EEC–Annex II. Species of community interest whose conservation requires the designation of special areas of conservation, Annex IV. Species of community interest in need of strict protection. Annex V. Species of community interest whose taking in the wild and exploitation may be subject to management measures).

Species or animal group	VLub n	VZach n	Period of collection, ecological category and other data
Family Mustelidae			
Eurasian otter *Lutra lutra* [120, 121]	5	1	2009–2014, semiaquatic and piscivorous (diet: seasonally up to 98% fish, 1.2% amphibians), body mass: 4000–8200 g; PL: native, partially protected species, increasing population; EU: annex II and IV
Badger *Meles meles* [122]	4	2	2009–2013, terrestrial and omnivorous (diet: up to 60–80% earthworms, 34% plant material, 28% vertebrates, 13% insects); PL: native game species, stable population; EU: annex V
Native medium-sized mustelids			
Pine marten *Martes martes* [123]	1	2	2010–2013, terrestrial and omnivorous (diet: seasonally 32% small mammals plus 3.5% carcasses, 11% birds, 27% plant material, 22% invertebrates); PL: native game species, stable population
Beech marten^4^ *Martes foina*	3	2	2009–2013, terrestrial and omnivorous (diet: about 28% mammals, 9% birds, 21% invertebrates, 35% plant material); PL: native game species, stable population
European polecat *Mustela putorius* [120, 124]	1		2010, semiaquatic and carnivorous (diet: seasonally up to 70% small mammals, 33% birds, 17% amphibians); PL: native game species, stable population; EU: annex V
Feral American mink *Neovison vision* [116, 125]	8		2009–2011, semiaquatic and piscivorous (^2^ diet: seasonally up to 62% fish, 56% mammals, 4–16% birds), alien game species (from North America), increasing population
Ranch American mink *Neovison vision*		7	2007, omnivores, body mass: 750–1500 g
Family Canidae			
Red fox *Vulpes vulpes* [126, 127]	1	13	2009–2014, terrestrial and carnivorous (diet: seasonally up to 60% small mammals, 21% carrion, ~ 20% birds, up to 26% plant material); PL: native game species, increasing population
Raccoon dog *Nyctereutes procyonoides* [125, 127]	8	7	2009–2014, terrestrial and omnivorous (diet: seasonally up to 51% plant material, 31% small mammals, 18% carcasses, 5% amphibians); PL: alien game species (from Eastern Asia), increasing population
Family Procyonidae			
Raccoon *Procyon lotor* [50, 125]	29		2010–2013, terrestrial and omnivorous (diet: ~44% mammals, 26% fish and frogs, 12% invertebrates, 2% plant material), range of body mass: 550–7200 g; PL: alien game species (from North America), increasing population
Raccoon dog *Nyctereutes procyonoides* [125, 127]	8	7	2009–2014, terrestrial and omnivorous (diet: seasonally up to 51% plant material, 31% small mammals, 18% carcasses, 5% amphibians); PL: alien game species (from Eastern Asia), increasing population

Only animals without visible head damage from car collisions or shooting were collected. The carcasses had been packed separately in plastic bags, frozen (-20°C) and stored in the laboratory until analysis. Brain samples were collected from 94 mesocarnivores, including 87 wild animals (63 and 24 from WMNP and VZach, respectively), and 7 ranch American minks (Table 1). In total, they represented 9 species belonging to three families (Mustelidae, Canidae, Procyonidae). Among them, alien feral species were American mink, raccoon (both from North America) and raccoon dog (indigenous to East Asia). Free-living carnivores from the WMNP were those killed in road accidents or caught in traps (mainly raccoons) under the program of limiting the population of alien mammal species in the park. Mammals collected in Vzach were either killed in road accidents or killed by hunters, who usually handed over skinned carcasses of these carnivores. In our work, pine marten (*Martes martes*), beech marten *M*. *foina*, and European polecat *Mustela putorius* were classified as one group: native medium-sized mustelids.

### Ethics Statement

The animals from the West Pomeranian Province were hunted by hunters who provided material for research in accordance with the Polish law. The animals from the Warta River Mouth National Park were killed in road collisions. Consent for this research was granted by the Director of the Warta Mouth National Park and the Regional Director for Environmental Protection in Gorzów Wielkopolski (WPN-1.6402.85.2011.KA).

### Metal analyses

From 12 raccoons, whose skulls were used in craniometric examinations, we collected only small samples (~3 g) from the back of the brain through the *foramen magnum* and only total Hg (THg) was determined in those samples. In the remaining raccoons and other carnivores, whole brains were extracted, in which Hg, Pb and Cd levels were determined. After the dissection of carnivore skulls, samples of the cerebrum (at least 10 g each) of the larger mammals and the whole brain of medium-sized mustelids were used in the study. The brains were dried at 55°C to a constant weight within 4–6 weeks, which made it possible to determine weight-based water content [33]. Dried brains were crushed in an agate mortar and concentrations of total mercury (Hg), lead (Pb), and cadmium (Cd) were assayed.

### Mercury

Concentrations of total Hg (THg) were determined using atomic absorption spectroscopy (AAS) at the Western Pomeranian University of Technology (WPUT) in Szczecin. The assays were run in an AMA 254 mercury analyzer (Altach Ltd, Czech Republic, detection limit ~0.01 ng Hg) in accordance with previously described methods [23]. Hg determinations were performed in samples weighing 0.1–0.3 g, usually in 2–3 replications for each brain, and statistical analyses used the means of the replications.

### Lead and cadmium

For the determination of Pb and Cd, samples were further dried in an oven at 105°C for 2–3 days. Both metals were determined at the WPUT in Szczecin. Digestion was performed in an Anton Paar Multiwave microwave oven (Anton Paar Ltd., Hertford, UK). About 1 g samples were weighed and transferred to pressurized quartz vials, into which 5.0 ml 65% HNO_3_ and 2 ml 30% H_2_O_2_ (both Merck Suprapur^TM^) were added. Vials were sealed with Teflon plugs, secured in mineralization bomb units, and placed in the microwave oven, with a temperature and pressure control system in each quartz vessel (for more details see [59]. Metals were assayed by inductively-coupled argon plasma optical emission spectrometry (ICP-OES) in a Perkin–Elmer Optima 2000 DV system. Lead and Cd detection limits for the system were 0.1 and 1.0 μg/L respectively.

Both of the analytical procedures were checked by determination of Hg, Pb and Cd concentrations in samples of two reference materials: dogfish liver (DOLT-4, Dogfish Liver Certified Reference Material for Trace Metals, Canadian Irradiation Centre, Laval, Quebec) and bovine muscle (8414 NIST, Bovine Muscle Powder, National Institute of Standards and Technology, Canada). Analytical results for the reference materials are presented in Table 2.

**Table 2:** Analytical results for content (mg/kg dw) of mercury (AMA 254) as well as cadmium and lead (Perkin–Elmer Optima 2000 DV) in certified reference materials DOLT-4 Dogfish and NIST 8414 Bovine Muscle Powder.

	DOLT-4			8414 NIST		
	Hg (n = 3)	Pb (n = 3)	Cd (n = 3)	Hg (n = 3)	Pb (n = 3)	Cd (n = 3)
Own results, OR	2.59±0.06	0.157 ±0.002	23.4±0.6	0.0054 ±0.0004	0.370 ±0.019	0.0127 ±0.0001
Reference value, RV	2.58±0.22	0.16±0.04	24.3±0.8	0.005 ±0.003	0.38±0.24	0.013 ±0.011
Recovery rate OR/RV (%)	99.6	98.1	96.3	108.0	97.4	97.7

### Statistical analysis

The concentrations of Hg, Cd, and Pb in the brain samples were expressed in dry weight (dw). A Kolgomorov-Smirnov test with Lilliefors correction showed that the distribution of Hg, Pb and Cd concentrations in the carnivores deviated from the expected normal distribution, so in the statistical analysis the comparison of the mean concentrations of the metals, non-parametric Kruskal–Wallis (K–W) or Mann–Whitney (M–W) tests were used when the number of means was respectively ≥3 or equaled 2. Results concerning mean metal concentrations were expressed as medians. Relationships between metal concentrations in the brain samples of each species (or NSM) as well as between body mass and brain metal levels of selected species were evaluated by calculation of Spearman correlation coefficient (r_S_). The differences in metal concentrations between animal species and the correlation coefficients in the raccoon and American mink were considered statistically significant at an alpha level of 0.05. All calculations were performed using Statistica 10.0 software (StatSoft Poland).

According to our results the average content of water in the brain in a multi-species group of mammals was almost 77% (76.7% in Eurasian otter and 75.4% of American mink from Poland). Therefore, in the comparisons of our results with those of other authors, it was justified to use 4.3 as a multiplier for wet weight to dry weight conversions.

## Results

Medians and ranges of the examined metals in the brains of the native and alien carnivore species from north-western Poland are presented in Table 3.

**Table 3:** Total mercury, lead, and cadmium (mg/kg dry weight) in the brains of carnivore mammals from NW Poland (n, number; Med, median).

Species		Hg	Pb	Cd
European otter *Lutra lutra*	n	6	6	6
	Med	2.444	0.156	0.026
	range	0.481–3.659	0.047–1.536	0.006–0.058
Feral American mink *Neovison vision*	n	8	8	8
	Med	3.964	0.208	0.055
	range	0.769–6.663	0.050	0.026–0.088
Ranch American mink *Neovison vision*	n	7	7	7
	Med	0.021	0.072	0.068
	range	0.019–0.038	0.068–0.443	0.059–0.305
Native medium-sized mustelids (*Martes martes*, *M*. *foina*, *Mustela putorius*)	n	9	9	9
	Med	0.106	0.212	0.029
	range	0.025–0.336	0.051–0.706	0.006–0.120
Badger *Meles meles*	n	6	6	6
	Med	0.124	0.305	0.035
	range	0.073–0.819	0.050–2.943	0.008–0.987
Raccoon *Procyon lotor*	n	29	17	17
	Med	0.145	0.470	0.026
	range	0.009–1.971	0.180–4.118	0.006–0.068
Red fox *Vulpes vulpes*	n	14	14	14
	Med	0.023	0.277	0.008
	range	0.011–0.236	0.183–33.51	0.007–0.034
Raccoon dog *Nyctereutes procyonoides*	n	14	12	15
	Med	0.150	0.184	0.036
	range	0.036–0.437	0.040–19.33	0.006–0.125

### Mercury

**Fig 2:**
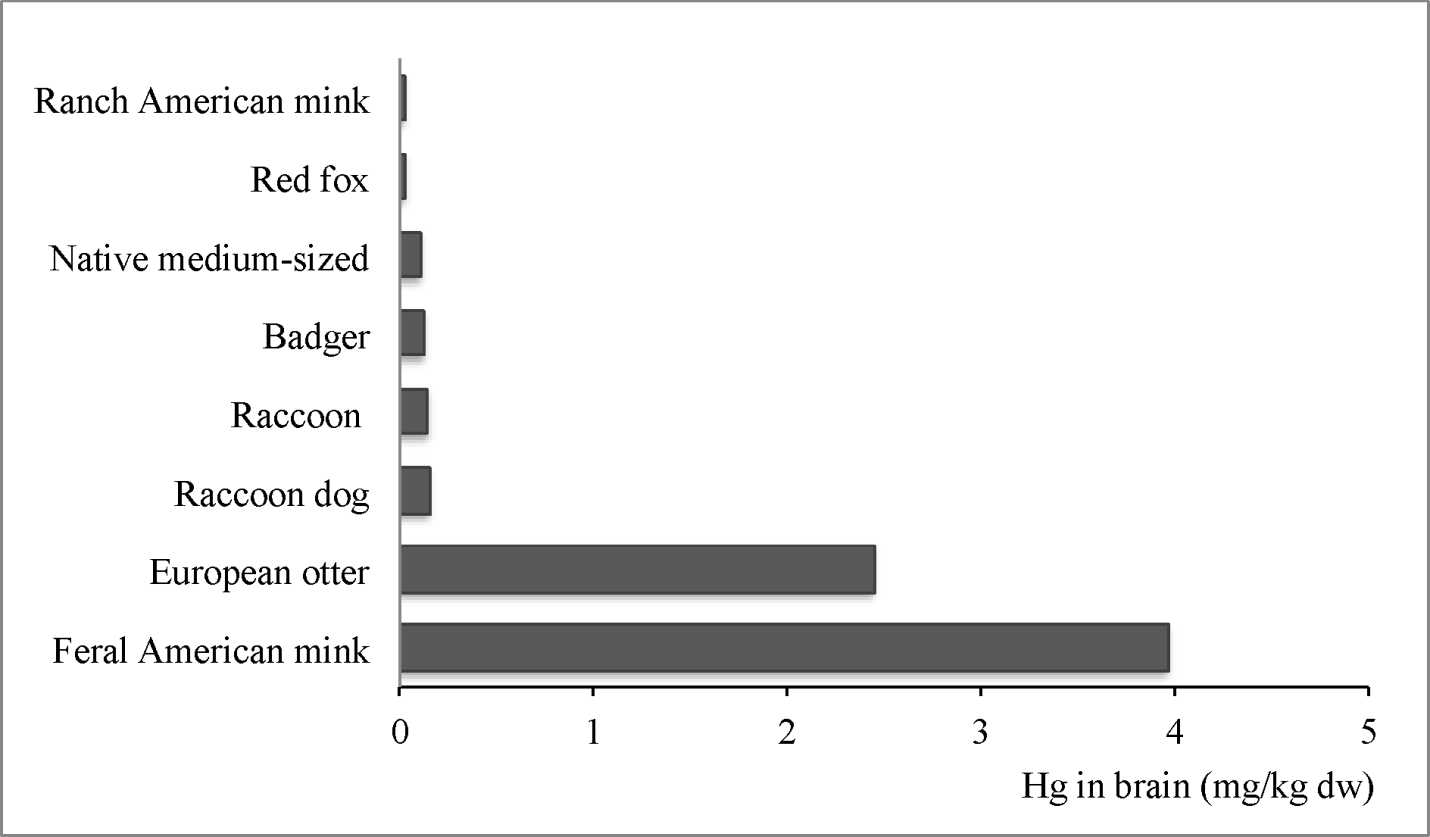
For several decades, this part of Europe has seen an increasing number of alien carnivores (raccoon, raccoon dog, American mink). Together with native species, they constitute a group of mammals with diverse food preferences, which is especially reflected in Hg levels.

The concentrations of mercury in the brains of the carnivores were in the range (0.009–6.663 mg/kg dw), with the highest medians in the piscivorous feral American mink and European otter (3.964 and 2.444 mg/kg dw, respectively). The lowest concentrations of this metal were found in the American ranch mink and red fox (median <0.025 mg/kg dw). Kruskal-Wallis tests (H = 53.04, p<0.0001, n = 93, df = 7) confirmed the presence of statistically significant differences between the compared brain Hg concentrations in carnivores from north-western Poland, in the following descending order: feral American mink > European otter > raccoon dog > raccoon > badger > native medium-sized mustelids > red fox > ranch American mink (Fig 2).

Hg levels in the brains of the piscivorous feral American mink and European otter were significantly higher than those of the other carnivores (p<0.001) and between each other (p<0.05). The lowest level of Hg was found in an adult male who preyed on fish from ponds near the town of Banie (VZach). Taking into account individuals of both these piscivorous species from the WMNP only, the difference in the concentrations of mercury in their brains was not significant (2.767 mg/kg dw in European otter vs 3.964 mg/kg dw in feral American mink). The raccoon and raccoon dog had significantly higher concentrations compared to the red fox (p<0.001) and the ranch American mink ranch (p<0.001).

**Fig 3:**
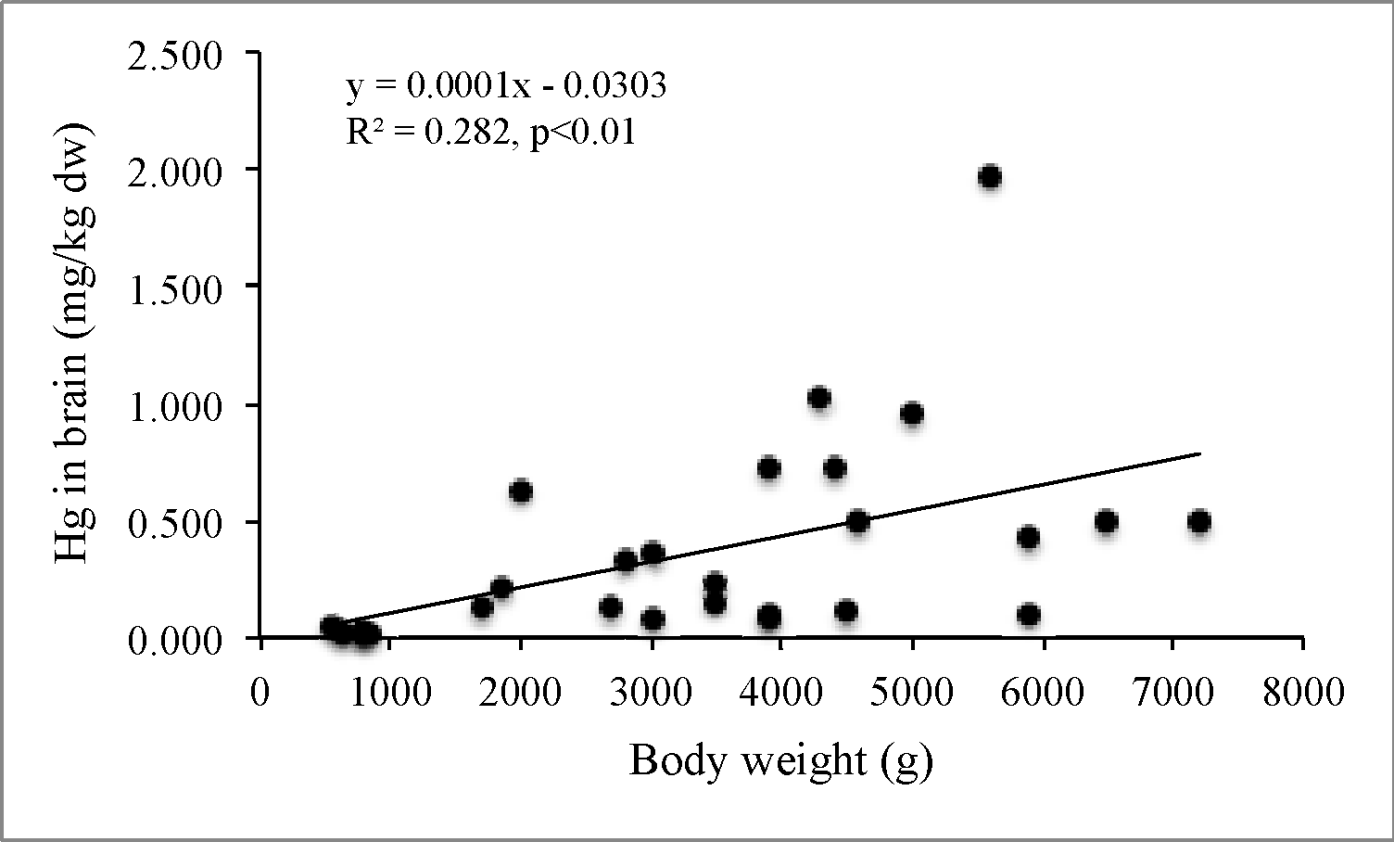
Analysis of data on raccoon, found frequently in the WMNP, shows a significant relationship between brain Hg levels and body weight in this partly piscivorous species.

Only in the case of the raccoon was there a sufficient amount of data (n = 29) on body weight and brain Hg concentrations. Analysis showed a significant correlation between these parameters (r_s_ = 0.603, p<0.001), as illustrated in Fig 3.

### Lead

The analysis of the Pb did not include 3 raccoon dogs in which the brain Pb level exceeded 100 mg/kg dw, indicating contamination resulting from hunting ammunition. Median Pb levels were in the range 0.072 to 0.470 mg/kg dw, with the minimum and maximum levels observed in the ranch American mink and raccoon, respectively (Table 3). Pb brain levels could be arranged in the following descending order: raccoon > badger > red fox > native medium-sized mustelids > feral American mink > raccoon dog > European otter > ranch American mink. Kruskal-Wallis tests (H = 15.64, p<0.05, n = 79, df = 7) showed the existence of differences between the groups. Pairwise comparisons (M-W test) of the study groups showed that brain Pb in the raccoon was significantly higher than in the European otter (p<0.01), native medium-sized mustelids (p<0.05), feral and ranch American minks (p<0.05). Moreover, the red fox had higher brain Pb content than the ranch American mink ranch (p<0.01). Other pairs of mesocarnivores did not differ significantly in brain Pb level.

In the brains of raccoon dogs from the WMNP, the Pb level was lower than in specimens from VZach (p<0.05), with medians of 0.073 mg/kg dw compared to 2.28 mg/kg dw, respectively.

### Cadmium

Brain Cd levels in the carnivores from north-western Poland ranged from 0.006 to 0.987 mg/kg dw. The lowest and highest medians were in the red fox and ranch American mink (0.008 and 0.068 mg/kg dw, respectively). The medians could be arranged in the following descending order: ranch American mink > feral American mink > raccoon dog > badger > native medium-sized mustelids > European otter > raccoon > red fox. The studied carnivores showed significant differences in brain Cd levels (K–W test: H = 29.42, df = 7, n = 82). Ranch and feral American minks had the highest brain Cd levels, differing significantly from the raccoon (p<0.01) and red fox (p<0.001). Also ranch mink had higher Cd levels in the brain than European otter (p<0.01), raccoon dog (p<0.01) and mid-sized mustelids (p<0.05). In raccoon dogs from the WMNP we found a higher brain Cd level (p<0.001) than in those in VZach (0.047 vs 0.008 mg/kg dw, respectively).

In raccoons from the WMNP, a significant inverse relationship between body weight and brain Cd concentration was observed, the Spearman correlation rank coefficient was negative (r_S_ = -0.541, p<0.05), with the brain Cd levels decreasing with the increasing weight and age of the raccoons.

We examined if there were any dependencies between the level of the studied metals in the individual groups of carnivores, and we found only a statistically significant correlation between Pb and Cd in the brain of the red fox (r_S_ = 0.758, p<0.01, n = 13).

## Discussion

European data on Hg, Pb, and Cd in the brains of wild and urban carnivores are very scarce [33,35,38,39]. For this reason a substantial part of this discussion relates to the North American reports.

### Mercury

Data from the 20^th^ century show that in many cases the unusual and strange behavior of domesticated and wild carnivores were caused by disorders of the nervous system. As was later found, disturbances were the consequence of anthropogenic pollution of aquatic and terrestrial ecosystems. Such behaviors usually preceded the disease in humans. The most famous example is environmental Hg poisoning in the Japanese Minamata Bay in the mid-1950s. Six years before the detection of Minamata disease in people, strange behavioral signs were seen in the local cats that had eaten the fish and shellfish with high amount of MeHg coming from the bay. The cats exhibited frenzied movements, throwing themselves against walls, or staggering as though intoxicated. Their mysterious behavior was called "dancing cat disease". Cats exposed to MeHg developed neurological and pathological changes similar to those observed in the humans with Minamata disease [60].

Another example of Minamata disease, and the cat dancing disease connected with anthropogenic mercury pollution coming from the local chlor-alkali plant, was found in the English-Wabigoon River system in Northwestern Ontario (Canada) in 1970. [61,62]. The brains of two cats from a village next to the English River, which had been fed fish from the river, had Hg levels at 16.4 and 6.9 mg Hg/kg. Along with the gradual decrease in mercury load, the English-Wabigoon River system saw a gradual re-colonization by piscivorous mammals. However, in the period 1983–1985 individual river otters still had THg brain >7 mg/kg ww (>30 mg/kg dw) [25].

Agriculture is another important source of Hg in environmental. In many well-developed countries of Western Europe, Japan and North America, pesticides containing organic Hg were used for the treatment of seed for much of the 20^th^ century [63], and reached the lakes and rivers in runoff. Firstly, this led to the intoxication of seed-eating birds and rodents, and then severe behavioral disturbances, decreased fertility and increased mortality in birds of prey and mammals from agricultural ecosystems. The dramatic increase in wildlife mortality on the agricultural background coincided with the use of Hg compounds in the pulp and paper industry and their release with wastewater into rivers and lakes. Large amounts of Hg coming from the mentioned sources were included into water food chains.

Hg intoxication of diurnal and nocturnal predatory birds, were first identified and described in Sweden, and later in other well-developed countries. After 1960, highly polluting industrial plants was growing with an increasing number of mercury-based chlor-alkali plants, mainly in Western Europe [64]. Borg et al. [65] described the abnormal behavior of a red fox similar to the “dancing cats”. In the fox’s mixed liver-kidney sample very high THg level (30 mg/kg ww) was detected. Also in other Swedish terrestrial carnivores (marten and polecat) large amounts of THg in such samples were found in the 1960s; unfortunately, Hg in their brains was not determined [65].

In biological samples THg is more often determined than MeHg, even though this Hg species is mainly responsible for neurological disorders in mammals. Data obtained from field studies and experiments show that mammals accumulate MeHg in the brain in amounts proportionate to the amount ingested [25,36,37,65,66]. The muscles of predators feeding on aquatic animals usually contain significant amounts of Hg, with the share of MeHg in THg exceeding 80% [25,34,67,68]. Inorganic Hg (InHg) has a long half-life in the mammalian brain, measured in years. The half-life of MeHg in the brain is much shorter than InHg and in primates (humans and the long-tailed macaque *Macaca fascicularis*) or raccoon it ranges from 1 to 4 months [34,69,70]. It seems that in the brain of terrestrial carnivores, for which fish, frogs and aquatic invertebrates constitute a considerable part of their diet, the dominant species of Hg is MeHg, constituting 70–100% of THg. The ability and effectiveness of MeHg demethylation in mammalian brains depends on many factors and differs between species. The diet of carnivores and MeHg content in their food varies depending on the species, season and region, and the amount of absorbed MeHg is indirectly reflected in the THg level in their brains [34,68,71,72].

In mammals InHg is mainly excreted with urine and feces (Clarkson and Magos, 2006). In MeHg elimination in fur-bearing carnivores probably hair may play the important role [34,73,74]. It is also conceivable that various molt pattern in otters, mink, and raccoon also contribute to differences in MeHg metabolism and Hg levels in their brains. In some mesocarnivores, brain THg increases with age, even at a relatively low Hg supply in the diet [33,66,68]. This is confirmed by our own observations on body weight and Hg brain levels in the raccoon.

### Mesocarnivores as mercury bioindicator candidates

In North America Hg is usually investigated in mink, river otter, and to a lesser degree in raccoon. Those mammals fulfill many criteria of good bioindicators, as they are numerous, common and may be legally hunted. Moreover, they have a relatively small home range, are long-lived and present in their habitats throughout the year, and their biology is well understood [21,25,75,76]. The mink has been used in experimental Hg intoxication and data on Hg brain concentration from laboratory and field investigations are quite rich in comparison to Pb and Cd (Table 4). In free-living river otters the highest THg brain concentrations (>30 mg/kg ww) were detected in Ontario (Canada) and USA (Virginia) before 1990 and after 2005, respectively [77,78].

**Table 4:** Comparison of total mercury, lead and cadmium concentrations (mg/kg) in the brains of wild and domesticated carnivore species (T, terrestrial; SA, semiaquatic; n, number; AM, arithmetic mean; SD, standard deviation; ND, not detected; the original levels expressed in wet weight are converted to dry weight using the 4.3 multiplier and given in brackets).

Species	Location and time	n	Mean concentration (AM±SD) and additional information	Range	References
Hg					
River otter, SA ^**a**^*Lontra canadensis*	USA, Wisconsin, 1972–1975	49	0.74±0.09 (3.18±0.39)^**c**^		[36]
River otter, SA	Canada, Manitoba, 1979–1981				[32]
	Winnipeg River	13	2.77	0.48–9.49	
	Wekusko (reference area)	17	0.85	0.04–1.71	
River otter, SA	Canada, Quebec, 1993–1994	11	0.72±0.21 (3.10±0.90)		[128]
River otter, SA	Canada, Ontario, 1994				[85]
	English River	4	3.25±3.40 (13.97±14.62)	0.23–7.15 (0.99–30.74)	
	Sudbury (reference area)	3	0.24±0.24 (1.03±1.03)	0.23–0.25 (0.99–1.07)	
River otter, SA	Canada, Ontario, 1994	41	0.28±0.13 (1.20±0.56)	max 0.46 (1.98)	[22]
River otter, SA	Canada, Nova Scotia, 1995				[129]
	inland	26	3.85^**d**^		
	coastal	40	1.57^**d**^		
River otter, SA	USA, Maine, 2000–2003	41	adult 0.51 (2.19)^**d**^	0.18–3.25 (0.77–13.97)	[130]
		19	juvenile 0.34 (1.46)^**d**^	0.06–2.01 (0.26–8.64)	
River otter, SA	Canada, 2001–2002 Nova Scotia	52	4.2±2.5	0.4–10.0	[30]
	Quebec	80	2.0±1.9	0.4–6.5	
River otter, SA	Canada, 2002–2004				[84]
	Nova Scotia	40	cerebral cortex 4.78±3.3		
			cerebellum 4.05±3.49		
	Ontario	23	cerebral cortex		
			1.23±0.36		
		26	cerebellum 1.05±0.36		
River otter, SA	USA, Wisconsin, 2003–2004	37	0.34±0.21 (1.46±0.90)	0.04–1.00 (0.17–4.30)	[68]
River otter, SA	USA, Wisconsin, 2009–2010	98	1.1±0.7	0.2–4.4	[131]
American mink, SA ^**b**^*Neovison vision*	USA, Wisconsin, 1972–1975	39	0.46±0.07 (1.98±0.30)		[36]
American mink, SA	Canada, Manitoba, 1979–1981				[32]
	Winnipeg River	62	2.95	0.17–7.41	
	Wekusko (reference area)	19	1.19	0.44–3.13	
American mink, SA	Canada, Ontario, 1983–1985				[85]
	English River	3	0.54±0.10 (2.32 ±0.43)	0.45–0.64 (1.93–2.75	
	Muskoka	4	0.55±0.10 (2.36±0.43)	0.46–0.66 (1.98–2.84	
American mink, SA	Canada, Quebec, 1993–1994	38	0.82±0.25 (3.53±1.07)		[128]
American mink, SA	Canada, Ontario, 1994	19	0.34±0.24 (1.46±1.03)		[22]
American mink, SA	USA, Maine, 2000–2003	90	0.44 (1.89)^**d**^	0.11–2.55 (0.47–10.96)	[130]
American mink, SA	Canada, Yukon, 2001–2002	30	0.22±0.16 (0.95±0.69)		[72]
American mink, SA	Canada, 2002–2004				[84]
	Nova Scotia	27	5.7±5.2		
	Ontario	10	1.4±0.6		
	Yukon Territory	11	1.2±0.8		
American mink, SA	USA, New York, Rochester Embayment Area of Lake Ontario (Area of Concern, AOC), 2004–2005				[132]
	in AOC/lakeshore	9	0.42±0.44 (1.81±1.89)		
	in AOC/inland	9	0.16±0.16 (0.69±0.69)		
	out AOC/lakeshore	9	0.30±0.16 (1.29±0.69)		
	out AOC/inland	9	0.19±0.15 (0.82±0.64)		
Polar bear, SA *Ursus maritimus*	Canada, Canadian Arctic, 2000–2003	24	0.24±0.07		[31]
Polar bear, SA	Denmark, Greenland, 1999–2001	82	brain stem 0.36±0.12	0.11–0.87	[133]
Raccoon, T *Procyon lotor*	USA, Wisconsin, 1972–1975	12	<0.02 (<0.09)		[36]
Raccoon, T	Canada, Ontario, 1973–1974	38	0.059±0.063 (0.254±0.271)		[134]
Raccoon, T	USA, California, 1993–1994 Sulphur Bank Mercury Mine			max. 1.15 (4.94)	[16]
	< 1 km	(?)	0.67±0.35 (2.88±1.50)		
	~10 km	(?)	0.15±0.08 (0.64±0.34)		
	11–16 km	(?)	0.63±0.56 (2.71±2.41)		
Raccoon, T	USA, Florida, 2000	11	0.286 (1.223)		[34]
Raccoon, T	USA, Tennessee, 2009–2010				[37]
	unexposed	10	0.0085 (0.037)^**e**^		
	exposed to coal fly ash in 2009	10	0.011 (0.047)^**e**^		
	exposed to coal fly ash in 2010	10	0.0165 (0.071)^**e**^		
Raccoon, T	Poland, WMNP, 2009–2011	8	adult 0.11^**e**^	0.08–0.49	[33]
		5	juvenile 0.01^**e**^	0.01–0.04	
Red fox, T *Vulpes vulpes*	USA, Wisconsin, 1972–1975	12	<0.02 (<0.09)		[36]
Red fox, T	Canada, Ontario, 1973–1974	40	0.057±0.062 (0.245±0.267)		[134]
Striped skunk, T *Mephitis mephitis*	Canada, Ontario, 1973–1974	8	0.099±0.079 (0.426±0.340)		[134]
American *marten*, *TMartes americana*	Canada, Quebec, James Bay	4	0.12 (0.52)	0.06–0.16 (0.26–0.69)	[25]
Egyptian mangoose *Herpestes ichneumon*	Portugal, 2011–2012	18	0.325^**f**^	0.022–1.4	[35]
Javan mongoose, T *Herpestes javanicus*	Japan, Amamioshima Island, 2004–2005	10	1.27±0.81 (5.46±3.48)	0.38–2.90 (1.63–12.47)	[29]
Pb					
Javan mongoose, T *Herpestes javanicus*	Japan, Amamioshima Island, 2004–2005	10	0.013±0.005 (0.056±0.021)	0.008–0.023 (0.034–0.099)	[29]
Dog, T *Canis lupusfamiliaris*	USA, Carolina		juvenile		[92]
	control	3	0.09±0.08 (0.39±0.34)		
	Pb-intoxictated	3	1.24±0.17 (5.33±0.73)		
Dog, T	Poland, Wroclaw, <1991	10	3–6 months 0.43±0.35 (1.85±1.50)		[38]
		10	1–7 years 1.03±1.37 (4.43±5.89)		
		10	15–21 years 0.98±0.96 (4.21±4.13)		
Cat, T *Felis catus domestica*	Poland, Wroclaw, <1997	35	cerebrum 0.144 (0.619)		[39]
			cerebellum 0.305 (1.311)		
Cd					
Javan mongoose, T *Herpestes javanicus*	Japan, Amamioshima Island, 2004–2005	7	0.005±0.003 (0.021±0.013)	ND-0.008 ND-0.034	[29]
Dog, T *Canis lupusfamiliaris*	Poland, Wroclaw, <1991	10	3–6 months 0.030±0.019 (0.129±0.082)		[38]
		10	1–7 years 0.041±0.039(0.176±0.168)		
		10	15–21 years 0.048±0.034 (0.206±0.146)		
Cat, T *Felis catus domestica*	Poland, Wroclaw, <1997	35	1 day– 20 years		[39]
			cerebrum 0.008 (0.034)		
			cerebellum 0.014 (0.060)		

^a^*Lontra canadensi*, previously *Lutra canadensis*

^b^*Neovison vision*, previously *Mustela vison*

^c^± standard error

^d^geometric mean

^e^median

^f^the value calculated on base of author’s data

Similar to the “dancing cats”, ataxia in the otter and mink from Saskatchewan (Canada) was observed as one of the clinical symptoms of Hg poisoning, although brain THg level in the mink (14 mg/kg ww) was much lower than in the otter [79]. In pathological studies of brains received from naturally Hg intoxicated carnivores like river otters, minks, the cats from the English-Wabigoon River system and lethally Hg poisoned in laboratory studies on minks, ferrets (domesticated form of polecat), river otters, dogs and cats, neuronal necrosis, astrogliosis, demyelination and vacuolation of neuropil are most frequently mentioned. The disturbances occur in the cerebral cortex (including the visual center) and cerebellum, which is responsible for balance. Lesions are less frequently detected in the midbrain, brainstem, and olfactory tract [44,62,80–82]. Moreover, due to an elevated brain THg in part of the experimentally intoxicated carnivores, researchers observed a deterioration or loss of vision, auditory impairment, and increased aggressiveness [44,80,82].

**Fig 4:**
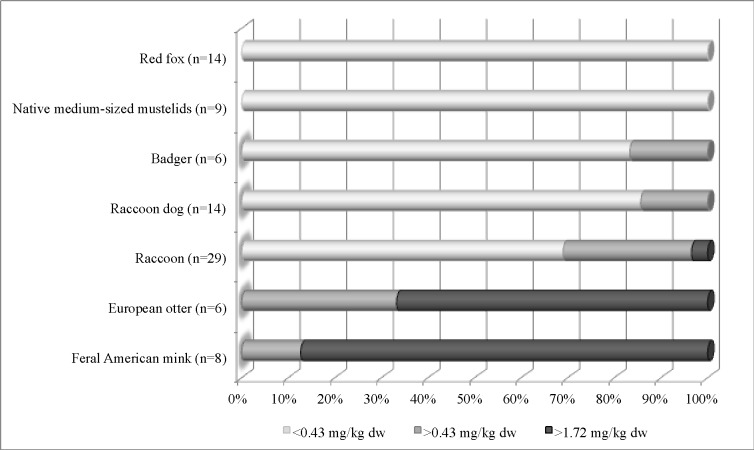
Krey et al. [83] have recently conducted extensive analysis of the brain Hg levels in a multi-species group of mammals, indicating relationships between various Hg concentrations and neurological changes. In our study, we used that analysis to classify the animals we studied based on brain Hg levels. Most often, the highest Hg levels, indicating neurobehavioural and/or neurochemical changes, were observed in mammals with some share of fish in their diets. These were minks, otters and raccoons, many of which were killed on roads.

Generally, in carnivores from field studies brain THg concentrations are much lower than the recorded levels in lethally Hg poisoned animals. However, even low Hg brain concentrations contribute to neurochemical and behavioral disorders that may adversely affect intra- and interspecies relationships. For most carnivores, brain THg thresholds and neurological effects are not known. Krey et al. [83] analyzed a considerable number of papers on brain Hg levels in laboratory, domestic and wild mammals and their neurological effects. On that basis, Krey et al. [83] proposed a THg threshold concentration for toxic endpoints and expressed them in wet weight (we also converted them into dry weight): clinical symptoms >6.75 mg/kg ww (29 mg/kg dw), neuropathological signs >4 mg/kg ww (17.2 mg/kg dw), neurochemical changes >0.4 mg/kg ww (1.72 mg/kg dw), and neurobehavioral changes >0.1 mg/kg ww (0.43 mg/kg dw). In Fig 4, we refer these brain THg threshold levels to the studied mammals by us. Among the 7 studied free-living carnivores, in the red fox and native medium-sized mustelid groups we found no specimens with brain THg >0.43 mg/kg dw. In 31% of raccoons it was 0.43 to 1.97 mg Hg/kg dw, but in most Eurasian otters and feral American minks THg brain concentrations were >1.72 mg/kg dw, which may indicate neurochemical and/or neurobehavioral changes. Importantly, almost all of the semiaquatic animals in our study were killed in road accidents. It is possible that the elevated Hg level resulted in impairment of their sensory perception and slower escape reactions, which could indirectly contribute to their death, and provide a bias in the results.

For comparison, in Table 4 we show the data on carnivore species, but taking into account only those papers in which average (mean or median) THg levels were given, and the number of specimens was ≥3. In many parts of eastern North America (Canadian provinces of Nova Scotia, Quebec, Ontario and the nearby US states including Main, New York and Wisconsin) data on THg have been collected since the 1970s, mainly in piscivorous mammals. In the past and at present, in areas not contaminated or slightly contaminated with Hg, the average levels of Hg in the brains of semiaquatic and terrestrial carnivores do not exceed 1.2 and 0.50 mg/kg dw, respectively [30,32,36,72,84,85]. In areas with a considerable anthropogenic Hg contamination, the brains of wild river otter and mink show mean Hg levels ranging from 2 to 5 mg/kg dw, similar to the Eurasian otter and feral mink from Poland (Tables 3 and 4).

Given the criteria of Krey et al. [83] it may be presumed many piscivorous otters and minks from Hg polluted areas of North America and Poland could experience neurochemical changes. Eurasian otter and feral American mink from the WMNP had similar brain THg levels (Table 3). This concurrence is also indicated by the research on river otter and mink in Canadian Nova Scotia, Ontario, Winnipeg and Mine in the U.S. [22,32,84]. This shows that Hg determination in samples collected from alien and very common feral American mink in many European countries may be a reliable alternative to the Eurasian otter, protected in the European Union and also rarely recorded in many member states. The usefulness of the Eurasian otter as a bioindicator of environmental Hg pollution is very limited.

In North America, besides river otter and mink, in other fur bearers brain Hg was only sporadically investigated. Papers on several carnivore species collected in one area are extremely rare [36]. In the 1970s THg was determined in 6 mammalian species (including river otter, mink, raccoon) from scattered sites in the highly industrialized state of Wisconsin, U.S. In the brains of those carnivores mean THg levels reached 0.74, 0.46, 0.08 mg/kg ww, respectively (Table 4). Those levels are similar to brain THg in two piscivorous species and raccoon from the WMNP in this study. In Wisconsin, the highest THg brain levels were noted in fish-eating carnivores which lived along the Hg polluted Wisconsin River [36]. About 30 years after that research, Strom [68] found less than half the THg brain concentration (0.34 mg/kg ww) in the river otter than in the specimens analyzed in the 1970s (collected from almost the same region). Strom [68] suggests that as the point-source discharges have now been eliminated or greatly reduced, the decreased level of THg in the river otters observed by him was likely the result of regional or geological factors.

However, as shown in other papers on North American river otter, mink, bats, and other wildlife and fish from the Swedish lakes, a few decades after the cessation of Hg pollution for industrial plants and the use of organic Hg compounds in agriculture, the residues still affect vertebrates and invertebrates that prey on animals that live constantly in water or periodically, such as amphibians and some insects [61,86,87].

It seems that THg analysis of mesocarnivores in our study, mainly from the WMNP, is another example of research in this field, as shown by the present and our previous research primarily on elements of the aquatic trophic chains [33,49]. In raccoon from the WMNP, whose diet is not more than 30% fish and frogs, we still detected elevated brain THg. Its average value was an order of magnitude greater than in unexposed raccoon from Tennessee (U.S.), similar to the Hg levels observed in these animals living in moderately Hg-contaminated areas (Wisconsin and California), but an order of magnitude smaller compared to the raccoon from highly Hg-polluted Florida wetlands (Tables 3 and 4). It seems that this species may be useful as a terrestrial bioindicator, especially of wetland and riparian ecosystems, similar to the raccoon dog, whose diet significantly depends on carrion and amphibians.

In our study, raccoon and raccoon dog had similar brain THg levels. Probably, Hg came from carrion (especially roadkill) and waterfowl, abundant in the WMNP. It can therefore be concluded that both these species, relatively numerous in this part of Europe, also mediate the movement of Hg from aquatic to terrestrial environments, but to a lesser extent than otters and minks. Badgers and native medium-sized mustelids had comparable brain THg levels to the raccoon and raccoon dog but their participation in Hg transmission into inland ecosystems is marginal due to their small number and density.

In our study, the increased brain THg levels in many carnivore species, mostly from the WMNP, indirectly reflected the current Hg pollution in north-western Poland. For over 50 years, a cellulose-paper plant operated in Kostrzyn and contaminated the Warta River with Hg before it underwent a renovation in the 1990s. The middle Odra River has been exposed to wastewater containing heavy metals (especially from a large Lubin-Glogow Copper Region, and chemical plant using Hg in Brzeg Dolny). In addition, the current Hg pollution of a considerable area in ​​north-western Poland is due to atmospheric deposition of Hg from various sites. This is indicated by the monitoring of heavy metal deposition carried under the European Monitoring and Evaluation Programme, EMEP. It is estimated that today in the countries covered by the EMEP, intercontinental transport contributes more than 65% of THg deposition [88]. This is also confirmed by European research on atmospheric deposition of Hg using mosses [89].

### Lead

In the past, in economically developed countries, lead was the major ecotoxicological concern, especially its highly toxic organic form, which was added to gasoline to reduce engine knocking. Elevated levels of brain Pb were reported in the inhabitants of large cities, urban dogs and cats, as well as many mammals living near roads with heavy traffic. Due to the high neurotoxicity, ethyl-leaded gasoline was eventually banned in the EU states (in Poland in 2003), the U.S., Canada, and Japan. In those countries, exposure to Pb has decreased dramatically since the 1980s, mainly because of the gradually increased use of unleaded gasoline [12,38,39,90].

The bioavailability of Pb is dependent on the form of Pb, quantity ingested, current nutritional status (including amounts of calcium and other metals, fat, and vitamins in diet), age of individuals, and their stomach acidity. Lead absorption at low values of gastric pH is more intense than at higher pH. Adult mammals absorb from 5% to over than 15% of ingested Pb, while fetuses, babies, puppies and pregnant females absorb nearly 50% of ingested Pb. Lead absorbed into the blood via gastrointestinal tract and lungs leaves the body in the urine, with a majority of ingested Pb excreted in the faeces without being absorbed. Organolead compounds are most readily absorbed, followed by Pb salts, then metallic Pb.

In the mammalian body, absorbed Pb is distributed to three major compartments: blood, soft tissue, and bone. Approximately 90–97% of Pb in tissue is skeletal. In the total human soft tissue, more than 90% of Pb is located in the liver and kidney, and small percentage is stored in the brain. Lead is taken by the brain when the blood level is quite low. Its retention in the brain persists after its blood levels fall. Therefore, if Pb intake is episodic, the concentration in the blood cannot be used as a measure of the amount of Pb in the brain.

Lead in the brain of long-lived mammals has a long half-life, in the order of years, with lead-induced brain injuries most likely being permanent [10,13,91,92]. The most common clinical signs of Pb-poisoning (plumbism) in domestic carnivores are vomiting, seizures, anorexia, hysteria, lack of coordination, and diarrhea. Most frequently, these are observed more frequently in cats than in dogs [93,94]. In dogs and other mammals, the toxic effects of Pb show as impaired visual discrimination or blindness, increases in aggression, impaired motor skills and learning behavior [14]. In environmentally and experimentally Pb intoxicated dogs, brain lesions are characterized by vascular damage and necrosis in the cerebral cortex, including the occipital lobe.

Clinical signs of plumbism were observed in experimentally orally Pb poisoned dogs fed a calcium-and-phosphorus-low purified diet with 100 ppm of lead acetate from age 6 to 18 weeks. In their brains segments, from 0.59 (~2.5 dw) to 2.36 (~10 dw) mg Pb/kg ww in the cerebellum and occipital lobe were detected, but the mean value of Pb brain was 1.24 mg/kg ww or 5.3 mg/kg dw [92]. In older (8 months) dogs (fed on a high-fat-low-calcium diet with a high dose of mixed lead salts) neurological signs were demonstrated when Pb level in the cerebrum was 3.3 mg/kg ww or 14.2 mg/kg dw [93]. It can therefore be assumed that the dog's puppies aged 5–10 months (and presumably in other carnivores) brain Pb concentrations >10–15 mg/kg dw indicates plumbism.

Little is known about subclinical signs, biochemical and behavioral changes and corresponding concentrations of brain Pb in humans and animals [91]. In control mammalian groups used in laboratory experiments and small mammals from reference area in field studies, brain Pb levels generally ranged from <0.10 to 0.50 mg/kg dw [92,95–97]. A concentration of <0.50 mg Pb/kg dw in the brain can therefore be considered as reflecting the background level.

However, even small, but chronic Pb pollution leads to accumulation in the brain of mammals. This is evidenced by data from the period when Pb coming from automobile exhaust and industry significantly polluted parts of developed countries, especially in urban environments. In the residents of Chicago (U.S.) during the 1970s, and Spaniards living in the zone of impact of a hazardous waste incinerator that did not have appropriate filters (data from 1998), brain Pb concentrations were 6.1 mg/kg dw, while in urban adult dogs from Wroclaw (Poland) Pb levels exceeded 4 mg/kg dw [38,95]. In cats from Wroclaw that spent much more time inside buildings, the brain Pb content was four times less than in the dogs (Table 3). These values are an order of magnitude higher than those recorded by us in the investigated canids (Tables 2 and 3). In Mexico, especially in the strongly polluted Mexico City, where leaded gasoline is still being used, brain Pb levels can exceed 20 mg/kg dw [98]. The cited works and our studies demonstrate the continuing problem of pollution with Pb and the need for its monitoring, including Pb in the mammalian brain.

Lead that enters the carnivore bodies with food may come from: 1) Pb bullet fragments found in the carrion and/or carcasses of game animals shot by hunters; 2) organic Pb incorporated in diet; 3) Pb (mainly in its divalent form Pb^2+^) contained in the soil, earthworms and dusted food; 4) the remnants of old flaking paints containing Pb, eaten by urban pets and synanthropic predators, such as fox and raccoon [11,14,42,94,99].

Comparing brain Pb levels indicating plumbism with our results we found 2/79 (2.5%) carnivores (red fox and raccoon dog) with Pb levels >15 mg/kg dw and 22/79 (29%) with Pb levels >0.5 mg/kg dw (including 9 individuals in the range 1.0–4.5 mg/kg dw: 3 raccoons, 2 raccoon dogs and single specimens of badger, European otter, feral American mink and red fox). In the other mammalian brains Pb level was <0.5 mg/kg dw. The studied mesocarnivores manifested a considerable diversity in terms of average brain Pb levels, with the distinctly higher Pb in the raccoon than in all mustelid groups.

Moreover, raccoon dogs from the WMNP, where hunting is prohibited, had a lower brain Pb compared to specimens from hunting grounds in the VZach (data not shown), suggesting that the elevated levels could have resulted from ingesting Pb from animals shot by hunters. In contrast to birds, lead intoxication from hunting ammunition has been poorly researched in wild mammals [14,42,45,100,101].

Among other things, absorption of Pb via the gastrointestinal tract depends on the acidic environment of the stomach. For this reason some carnivores, such as canids and raccoons, with low gastric pH (about 1–3) may absorb more Pb from the diet than piscivorous carnivores with a higher gastric pH [102,103]. This is indirectly shown by analyses of livers of European otters from Hungary and wild minks from Virginia, U.S. In those piscivores, Pb was detected in 49% and 6% hepatic samples, respectively [104,105]. Diters and Nielsen [100] found a wide Pb range (1–35 mg/kg ww) in livers in North American raccoons. Based on our research and other papers, it may be concluded that the brain of this omnivorous predator, similarly to the red fox and raccoon dog, may be used in the indirect assessment of Pb pollution in rural and suburban environments, similar to the brain of the domestic dog in urban habitats. In addition, these two species make it possible to perform comparative analyses not only on the local but even on the intercontinental scale, given a suitable body of data.

### Cadmium

Cadmium is absorbed via the gastrointestinal tract and lungs, and is mainly accumulated in the kidneys and liver. Bone Cd concentration is lower than in the organs but its negative effects, known as Itai-Itai disease in humans, have been documented in several investigations on animals. The distribution of Cd between body parts differs markedly depending on the chemical species of Cd, dosage and length of exposure. In people (and probably in other long-lived mammals) the biological half-life of Cd is very long, up to 30 years [40,106,107]. In dogs the half-life of Cd was estimated at about 1 to 2 years when Cd was present in the diet at 1 to 50 mg per day [108]. From the ingested food, which plays the most important role in Cd uptake in animals, mammalian blood absorbs from ~1% up to 5–6% of Cd; in dogs ~3% [106,107,109]. In wild mammals airborne Cd probably does not play a significant role, but it is important in occupational exposure of some workers, active and passive tobacco smokers (tobacco leaves accumulate high levels of Cd from the soil) as well to the domestic dogs and cats [106,110]. Absorbed Cd is excreted in the urine with the remaining portion removed with feces [106,107,108]. Klaassen and Kotsonis [111] suggest that in the dog, rat and rabbit, biliary excretion is the main route for Cd elimination.

Only a very small part of absorbed Cd is deposited in the mammalian brain. Its damage has been observed in laboratory rodents eating or drinking inorganic Cd. However, this area of research lacks reliable information which could indicate how much of the ingested Cd results in the impairment of nerves or brain of people or other non-rodent mammals [10]. During neonatal development, mammalian brains may absorb more Cd as it is able to readily pass to the fetus via the placenta and has also been detected in milk during lactation. Moreover, the brains of intensely developing organisms do not have a fully developed choroid plexus which in adult mammals accumulates large amounts of Cd and is also seriously affected. Apart from this, Cd can be uptaken from the mucosa of the nose and/or olfactory pathways into the central nervous system. Based on animal models it has been shown Cd is more toxic to newborns and the young than adult rodents. Perinatal exposure to Cd reduces the brain weight of pups and inhibits the activities of enzymes in brain. This toxic metal induces neuronal death and is responsible for apoptosis and necrosis in the developing brain [28,112]. Mice treated with high doses of Cd chloride (100 mg/l) in drinking water experienced histopathological changes in their brain including congestion of blood vessels, necrosis, focal gliosis, and the atrophy and necrosis of pyramidal cells [26]. Chronic exposure to Cd causes brain disorders, e.g., olfactory dysfunction and memory deficits, as shown in laboratory rodents and in persons chronically occupationally exposed to Cd [27,28,106].

Cadmium in the brain is rarely determined in laboratory rodents but the Cd level in the control groups usually varies a lot, sometimes by 1–2 orders of magnitude. In control rats used by Gupta et al. [112] brain Cd concentrations in 7, 14, and 21 day old pups were 0.15, 0.18, 0.19 mg/kg ww, respectively. In another study much lower but increasing values of brain Cd were found in control rats: 0.03 and 0.04 mg/kg ww for 7 and 21 day old pups, respectively [113]. In 5–6 month old control rats, in all the analyzed brain parts (cortex, hippocampus, hypothalamus, striatum and cerebellum) Cd levels were similar, at <0.009 mg/kg ww [114]. In yet another study, adult rats from the control group had mean brain Cd of 0.071 mg/kg ww [115]. The differences in brain Cd levels were due to differences in the Cd content in commercial feed given to the rats in the laboratories [115], demonstrating a direct link and readiness to accumulate this metal.

Available data on brain Cd in non-experimental animals are extremely scarce (Table 4). In domestic cats from Wroclaw (Poland) Cd brain level was <0.015 mg/kg ww but in the dogs from that city, in the area of impact of the Lubin-Glogow Copper Region, brain Cd level was one order of magnitude greater [38,39]. The brain of a wild terrestrial carnivore from Japan, the Javan mongoose, contained on average 0.005 mg Cd/kg ww, and the range was from below detection limit (DL) to 0.008 mg Cd/kg ww [29].

Given these limited data, it can be concluded that Cd enters the brains of free-living mammals at very low or undetectable amounts. Therefore, establishing background Cd levels for the mammalian brain is very difficult at the current state of knowledge. Taking into account previous studies on laboratory rat pups investigated by Goncalves et al. [114], and on cats and wild carnivores, one can cautiously suggest that the background Cd level in the brain is below 0.009 mg/kg ww or 0.04 mg/kg dw. In the mesocarnivores examined by us brain Cd concentrations were low and median values ranged from 0.008 to 0.068 mg/kg dw.

Analysis of the results showed that the studied mammals accumulate Cd in their brains in varied amounts, depending on the diet and degree of environmental pollution with Cd. The highest medians (>0.04 mg Cd/kg dw) were found in the brains of feral and ranch American minks (0.055 and 0.068 mg/kg dw). However, the highest brain Cd (~1.0 mg/kg dw) was found in the badger, an animal which consumes large amounts of earthworms. In addition, we found that the raccoon dogs from the two analyzed areas differed in brain Cd levels, with the lower concentration found in specimens from VZach. This may indirectly attest to the greater Cd contamination in the Park than the VZach, which is also confirmed by a study on Cd levels in moss and soil samples [89].

Research on laboratory rats and urban dogs shows that Cd concentration in the brain increases with age [38,112,113]. In the raccoon from the WMNP we found a significant negative correlation between brain Cd level and body weight. This may be related to penetration of a certain amount of Cd from the mother's body to the brain of fetuses in the perinatal period, which has been shown in other mammalian species [114]. Furthermore, it is likely that compared to adults, young raccoons initially eat more of the easily accessible and more Cd-rich food such as plant material, earthworms and ground insects [116,117]. In adult rats Cd permeates to the brain to a much lower extent than in growing animals, which is related to the maturity of the blood-brain barrier [28,113,114]. Probably, this is why the adult brain has a lower Cd concentration than in the initial months of postnatal development. One also cannot exclude the possibility that in adult raccoons, Cd is gradually removed from the brain, but this requires further research.

### Toxic metal interactions

Wildlife and humans are exposed to a cocktail of toxic heavy metals in the environment. However, in mammals, including carnivores, research usually concerns individual metals from this group. At the same time, as shown by experiments on mice, mixtures of metals possess higher toxicities compared to individual metals. Exposure to low doses of three metals: Pb, Hg, and Cd reduces brain mass and induces structural lesions in mice, as well as increases oxidative stress in the brain [118]. Those authors and Jadhov et al. [119], who tested chronic exposure of a cocktail of 8 heavy metals (including Hg, Pb, Cd) in rats, suggest that interactions in toxic metal mixtures are basically synergistic in nature and can induce necrosis in some organs, including the brain. In our study, most of the studied mesocarnivore species from north-western Poland were generally exposed to low levels of these toxic metals. In the red fox we did observe a positive and significant correlation between brain Pb and Cd concentrations, which to a certain extent supports the propositions of Jadhov et al. [119] and Cobbina et al. [118].

## Conclusions

Our research on 94 brains from 9 native and alien species of mesocarnivores is unique and provides important information in the field of ecotoxicological studies. Analysis of the toxic metals in animals from north-western Poland, including protected areas near the Poland-German border, showed that depending on the diet and degree of environmental pollution, carnivore brains accumulated Hg, Pb and Cd in diverse amounts. In Europe, the brains of alien species such as the semiaquatic feral American mink, terrestrial raccoon, raccoon dog and native red fox, can be used in bioindicator research due to their common occurrence and numerous populations with well-known biology.

The Warta Mouth National Park is significantly polluted with heavy metals, especially mercury, as a result of historical and present Hg deposition from human activity. The highest and comparable mean brain Hg levels were found in the piscivorous Eurasian otter and alien feral American mink from a floodplain area of the Odra and Warta rivers. This shows that in th/e European Union, Hg-bioindication may used alien feral American mink, numerous in many European countries, instead of the rare and protected European otter. Moreover, our results show that the transfer of Hg from aquatic to terrestrial ecosystems is also mediated by raccoons and raccoon dogs, numerous in this part of Europe.

Carrion with hunting ammunition is likely to be an important source of Pb for omnivores and partial scavenger species, the especially raccoon dog. Raccoon dogs living in the heavy metal polluted national park were more exposed to ingested Cd than others. Importantly, based on our own results and literature data, we for the first time suggest background levels of brain Pb and Cd in mesocarnivores of <0.50 and <0.04 mg/kg dry weight, respectively.

Suggestions of other researchers that interactions in toxic metal mixtures are basically synergistic in nature and can induce damage in the brain in laboratory mammals (as shown in a few papers) and the correlation found between Pb and Cd concentrations in red fox in this study, are a significant premise to intensify research on wildlife in this area.
